# Examining the online approaches used by hospitals in Sydney, Australia to inform patients about healthcare associated infections and infection prevention strategies

**DOI:** 10.1186/s12879-017-2899-2

**Published:** 2017-12-21

**Authors:** J. Park, H. Seale

**Affiliations:** School of Public Health and Community Medicine, University of New South Wales, Level 2, Samuels Building, Sydney, NSW Australia

**Keywords:** Patient information, Internet, Hospital website, Patient involvement, Healthcare associated infections, Healthcare acquired infection, Infection control and prevention, Hand hygiene, Health literacy

## Abstract

**Background:**

Provision of information plays a critical role in supporting patients to be engaged or empowered to be involved with infection prevention measures in hospitals. This explorative study evaluated the suitability, readability and accessibility of information on healthcare associated infections (HCAIs) and infection prevention strategies targeted at patients from the websites of 19 acute care public hospitals in Sydney, Australia.

**Methods:**

We included hospitals with greater than 200 beds in the sample. We examined online information targeted at patients on HCAIs and infection prevention and compared it using the Suitability Assessment of Material (SAM) and Simple Measure of Gobbledygook (SMOG) readability formulas for suitability, readability and accessibility.

**Results:**

Thirty-six webpages were identified as being relevant and containing information about HCAIs or infection prevention. Based on the SAM/SMOG scores, only three webpages were found to be ‘superior’. Many of the webpages scored poorly in content, literacy, graphics, learning stimulation and cultural appropriateness. In comparison, most of the webpages scored well in the layout and typography. The majority (97%) of the materials were written at a level higher than the recommended reading grade level. Lastly, the websites scored poorly on the ability to locate the information easily, as messages about HCAIs/infection prevention were usually embedded into other topics.

**Conclusion:**

While providing information online is only one approach to delivering messages about infection prevention, it is becoming increasingly important in today’s technology society. Hospitals are neglecting to use best practices when designing their online resources and current websites are difficult to navigate. The findings point to the need to review patient information on HCAIs regarding suitability, readability and accessibility.

## Background

While there are a range of interventions recommended and implemented to avoid healthcare associated infections (HCAIs), evidence suggests that hand hygiene (HH) is the single most important measure to prevent the transmission of HCAIs [1–3]. To achieve high rates of compliance with strategies such as HH, it has been advocated by the WHO and other agencies that the patient must be an active participant in the infection prevention process. Patients who are actively engaged in their care are more likely to identify and mitigate potential risks by asking healthcare workers (HCWs) questions about their HH practice. The Australian National Safety and Quality Health Service (NSQHS) Standards supports the idea of patient empowerment in relation to the clinical benefits associated with decreased rate of HCAIs [4]. However, it is well recognised that empowering patients to ask HCWs about their HH is challenging [5–10].

The provision of information on HCAIs could positively influence the patients’ engagement in infection control strategies [11, 12]. Previous studies have shown that patients who receive information on HCAIs demonstrate better understanding about the pathogens; risks etc. and are more actively engaged in infection control and prevention measures compared to those who had not received any information [13, 14]. However, it is important to recognise that it is not enough to just provide information. The strategy used to deliver the information is also important. For example, it has been previously shown that providing patients with only verbal information is ineffective as the retention of this information can be short-lived, whereas the provision of written information is thought to be more beneficial [15, 16].

Under the Health Act (2006), NHS Trusts have a duty to provide information about the risks of HCAI, a requirement which exists in other countries such as France and many states of the USA. In Australia, performance information is provided on the myhospitals.gov.au website. The website includes data on HH performance, as well as on rates of HCAI *Staphylococcus aureus* bloodstream infections and allows for comparison between hospitals. Even though improvements have been made to the availability and transparency of information provided about HCAIs, this does not necessarily equate to consumers engaging with the information. Numerous studies to date have documented that the lay media is still the public’s primary source of information about HCAI [17–19]. It is perhaps not surprising that this leads to misguided perceptions of risk given the alarmist nature of these sources [20].

Considering the fact that 60% of Australian adults have low health literacy and patients’ knowledge of HCAIs is limited [21], assessing the information available for patients on HCAIs is an important step in ensuring the safety and quality of patient care. Information will only be useful if it is understood and used by the public. The internet is selected as a source of information as it is identified by the public as the second most used information source following their doctors’ advice [22]. People with low health literacy are more likely to seek health information from computer-based health information systems [22]. Therefore, it is important to review the suitability, readability and accessibility of information aimed at informing and engaging patients about HAI prevention and control strategies.

A literature search was undertaken to locate previous studies which have focused on the development and delivery of patient information around infection control and prevention strategies. However, there has been little research done to date evaluating infection control information brochures or online material for patients. Therefore, this study will be the first to explore the approach taken in Sydney, Australia to inform hospital patients about infection control and HH. We acknowledge that in the hospital setting a multimodal approach is often taken to inform patients about infection control, however for this study the focus is on information available online from official public hospitals’ websites.

## Methods

### Identifying target hospitals

An Internet search was conducted using the Google search engine to identify all public tertiary referral hospitals (with >200 beds) in Sydney. Myhospital.gov.au (https://www.myhospitals.gov.au/) and Local Health Districts (LHDs) and Specialty Networks (http://www.health.nsw.gov.au/lhd/pages/default.aspx) were used to confirm the list of public hospitals found from the Google search. Mental health facilities and Justice Health were excluded from the list. The remaining excluded sites included private hospitals, day surgery’s and specialist centres (i.e. heart clinics, endoscopy centres etc.). We purposefully targeted the large tertiary referral hospitals in Sydney as we believe that they would benefit most from the findings from this study due to their high patient numbers and continued burden of healthcare associated infections. We also purposefully excluded reviewing the smaller public hospitals as it was assumed that their websites would be controlled by the same area health services as the tertiary referral hospitals and so the content would be the same.

### Data collection

A search of the websites of all public hospitals (with >200 beds) was conducted to locate any resources that appeared to be targeted at patients (current/future) and which focused on ‘HCAIs’, ‘infection control’ and ‘prevention measures’. A range of different words and phrases were used to try and locate relevant webpages and or other relevant files/resources (i.e. downloadable brochures, videos). The search terms included: ‘infection’, ‘hand hygiene’, ‘hand’, ‘wash’, ‘healthcare acquired infection’, ‘HCAI’, ‘prevention’, ‘prevention and control’, ‘patient information’, ‘germs’, ‘bug’ and ‘clean’. Any webpage/resource containing those search terms was reviewed for relevance by JP. Hospital policies and staff guidelines found in the search were excluded.

### Data analysis

All information identified in the search was evaluated using two assessment methods: Suitability Assessment of Material (SAM) and Simple Measure of Gobbledygook (SMOG) readability formula. Both SAM and SMOG are validated and recommended tools [23–26]. Initially the ‘Clear Communication Index Score’ was considered but it was found to not be suitable to evaluate video/audio material. SMOG was recommended for evaluating patient education materials and was incorporated into SAM [27].

### SAM (Suitability Assessment of Materials)

All information found from the search was evaluated and scored using the detailed evaluation criteria provided in Teaching Patients with Low Literacy Skills [27]. SAM has 22 factors that are grouped under six categories: content, literacy demand, graphics, layout and typography, learning stimulation, motivation and cultural appropriateness. Each factor is given a score of either 0 (not suitable), 1 (adequate) or 2 (superior) depending on how the material meets the criteria. Some factors were not applicable such as reading grade level for video material, which marked as ‘N/A’ on the scoring sheet. As instructed, 2 points for each N/A items were subtracted from the 44 total when calculating scores. A sum of each factor was divided by the highest possible total score. The figure, then, was multiplied by 100 to get the percent score. The percentage scores were interpreted as ‘superior (70–100%)’, ‘adequate (40–69%)’ or ‘not suitable (0–39%). JP was responsible for identifying, reviewing and scoring the located webpages/resources. All findings, including discrepancies were discussed with HS.

### SMOG (Simple Measure of Gobbledygook)

Written text from the resources was cut and pasted into the readability formula website (http://www.readabilityformulas.com/free-readability-formula-tests.php) for SMOG score. Grade level is an estimated level of education that people require understanding the given written information. 5th grade level is an ideal reading level marked as ‘superior’, between 6th -8th grades is ‘adequate’ and 9th grade and above is deemed ‘not suitable’.

## Results

A total of 19 acute care public hospitals (with >200 beds) under eight Local Heath Districts (LHDs) in the Sydney region were identified from the search. Most of the hospitals (14/19) were located within 50kms of Sydney’s central business district (CBD).

From the websites of the 19 hospitals we could locate 36 webpages containing information or other resources (links, videos etc.) that were relevant. Of the 36 webpages, 29 contained written information on the actual webpage about HCAIs or infection prevention. The remaining sites had either a downloadable PDF (2/36), a link to an external site (1/36) or video clip (4/36).

More than one-third of the hospitals (8/19, 42%) offered a single electronic resource on HCAIs or HH, whereas others had between 2 and 4 electronic resources available for patients (Table 1). In a high proportion of cases (29/36) the webpage was not exclusively dedicated to information about HCAIs and HH. Instead, messages about infection prevention were embedded into webpages focused on providing general information about hospital admissions or as part of a welcome message to patients attending the hospital. Only 7/36 webpages had explicitly dedicated areas of the site to information about HCAIs, HH or other infection prevention strategies.

**Table 1:** Mean suitability and readability scores for each hospital

Hospital	# of resources	Mean SAM	Mean SMOG	Mean SAM by %	Overall suitability rating
1	3	13	11.47	65	Adequate
2	3	15.33	10.87	38.33	Not suitable
3	3	14.67	11.9	36.67	Not suitable
4	3	15.67	11.37	39.17	Not suitable
5	2	26	10.5	61.03	Adequate
6	1	20	12.9	50	Adequate
7	2	26	10.5	61.03	Adequate
8	1	23	9.4	57.5	Adequate
9	1	24	NA	66.67	Adequate
10	4	23.75	9.77	58.86	Adequate
11	2	15.5	10.25	38.75	Not suitable
12	2	15.5	10.25	38.75	Not suitable
13	1	9	8.9	22.5	Not suitable
14	1	9	8.9	22.5	Not suitable
15	2	26.5	8.3	66.25	Adequate
16	2	15.5	9.6	42.82	Adequate
17	1	18	8.8	45	Adequate
18	1	18	8.8	45	Adequate
19	1	16	9.5	40	Adequate

In many cases, it was difficult to locate the information about HCAIs and infection prevention on the hospital website. Often the information was embedded into other material so that to locate the relevant material you were required to click through other pages/sites. Focusing on the webpages that contained infection prevention information embedded into other general hospital information, only half (16/29) have clear subheadings. Search boxes offered on the webpage had to be utilized to find any relevant information. However, most of the search results returned irrelevant information for patients such as policies or hospital guidelines.

26/36 of the webpages located included information focused on visitor’s behavioral changes to reduce the risk of HCAIs such as performing HH, respiratory etiquette techniques and encouraging hospital visitors to stay away if they are unwell. Only one-third (12/36) mentioned patient empowerment strategies in infection control strategies and less than one-third (10/36) of the information addressed the rationale for HH by patients. Only 3 explained what a HCAIs is.

More than one-third of hospitals (8/19) had a modified web version for mobile use. However, the mobile version did not support all the graphic information. One of the webpages could not be opened in a certain browser.

Of the four video clips found, two contained general information relevant to a hospital patient staying at the hospital. The remaining two videos focused on the importance of HH but none addressed HH from the patient perspectives. The videos ranged in length between 30 s and 16 min 36 s. Only two included supporting information about the video, and none had written captions or information available in languages other than English. The video that went for 16 min 36 s included general information about attending the hospital including location of the hospital, visiting hours, discharge planning process, services for Aboriginal and/or Torres Strait Islanders, interpreter service, smoke free policy, use of televisions, telephones and mail service, managing medications, etc. Patients had to watch over half of the video before they came across any information about infection prevention. While most videos encouraged patient and visitor HH, only one included a demonstration of the correct way to HH. None of the videos included any background information about HCAIs. Lastly, only one video made mention of the role patients can have in encouraging staff members to HH.

Based on the SAM and SMOG scores, 11/36 webpages were found to be ‘not suitable’ for patients. While 22/36 of the webpages were found to be ‘adequate’, only 3 were ‘superior’. The interpretations of SAM scores are summarized in Table 1. The highest SAM score (%) was for a webpage that included a link to a HCAIs Consumer factsheet which was located on an external site (which had been developed by the National Health and Medical Research Council) and the lowest score was observed for websites which only included a single sentence of HH information which was embedded into the welcome message. The results of SAM are presented below according to the factors listed on the tool.

### Content

Most infection prevention messages (29/36) were embedded into general information and only half had subheadings relating to infection control or HH. Although the majority contained information about desirable behaviors of patients to reduce the risk of HCAIs, the amount of information mentioning HH or HCAIs was minimal. Thus, most of the resources (25/36) scored 1 or below for the ‘content about behaviour’.

As a wide range of topics were usually addressed within a single resource, the scope of information directly relating to the HH or HCAIs was limited. Additionally, the placement of the target information was commonly at the bottom of the webpage which negatively influenced the reading time allocated. Only 1 site included a summary of key points.

### Literacy demand

Of the 36 resources, 33 were assessed for the SMOG (Simple Measure of Gobbledygook) readability test including one video. The mean SMOG score (10.32) was above 9th grade level (collage level), which is 4 grades higher than the recommended level. Only 1 resource was found to be ‘adequate’ (6 to 8 grade) with a readability score 6.9. None of the resources met the superior criteria, which is 5th grade level or lower.

On a positive note, an ‘active voice’ (i.e. “wash your hands”) was used in more than half of the resources (62.5%) which focused on addressing desirable behaviors for infection controls. Very little jargon was used and technical terms relating to definitions or categories of HCAIs were explained. About 50% of the sites, provided the context before any new information was given such as “*There are a number of things you can do to reduce the risk of infection….to wash your hands…with soap and water or use an alcohol-based hand rub*”.

However, ‘road signs’ that help readers’ thought process were not utilized in most of resources. As the information relating to HCAIs or HH was generally embedded into other information with headings such as ‘visitor information’ or ‘during your stay’, the road signs were not directing readers to the information addressing HCAIs or HH.

### Graphics

Of the 36 resources, most (22) contained graphics but only two contained graphics that directly related to the purpose of information on HCAIs, and which were deemed to be attractive. The rest of the graphics (20/22) showed a patient being looked after by HCWs or HCWs in uniforms but did not clearly indicate the intention of the messages and were generic stock photos. More than one-third (14/36) did not contain any graphics. Thus, scores for the relevance of illustrations were rated low. The use of a chart or graph was minimal (3/36).

### Learning stimulation, motivation

Only a small number of the sites (5/36) used the format of ‘question and answer’ to stimulate learning. Only 1 of the sites met the superior level for this criterion by enabling readers to respond to problems/questions presented, while the remaining four had some aspects of passive interaction (question and answer format).

Most of the sites (33/36) contained some form of instruction on how to conduct HH (either with soap and water or alcohol-based hand rub), when and where to perform HH, and other ways to reduce infections. Only 3 sites did not contain any behavioral instructions.

Given that “chunking” of the information was often used with or without descriptive headings and generally without the use of question-answer response format, achieving self-efficacy by solely reading the information provided may prove difficult for patients. Hence, the overall rate for evaluating motivation (self-efficacy) is low with 20.83%.

### Cultural appropriateness

Logic, language and experience (LLE) in the materials were assessed against the LLE of the intended audience. None of the sites provided information in any other languages and most pictures included on the sites were of Caucasians.

## Discussion

In this study, we have evaluated the suitability, readability and accessibility of the information on HCAIs and HH for consumers from the official websites of 19 acute care public hospitals (> 200 beds) in Sydney. We found that only 3 webpages were ‘superior’ whilst 91% were ‘adequate’ or ‘not suitable’ based on the scores for the overall suitability test. Locating the relevant information currently takes time and effort. Many of the webpages scored poorly in content, literacy demand (reading grade level), graphics (use of graphics and its relevance), learning stimulation (interaction used) and cultural appropriateness. However, they did score well in the layout and typography.

Information focused on HCAIs and HH was mostly collated with general information about ‘staying at the hospital’ (i.e. about patient rights, using the entertainment system, etc.). In most cases, there was little in the way of headings/subheadings so searching for the information on HCAIs and HH required multiple clicks through other pages/sites. Information provided in an obscure way could be a barrier for people especially those with reading and IT difficulties [28].

Nearly all the sites (97%) reviewed were graded as ‘not suitable’ according to the SMOG readability test. The information being presented on HCAIs is currently too difficult for people to understand and act upon, especially for those who speak English as a second language and have lower health literacy. This is similar to findings from previous studies that have evaluated readability of patient information across other specialties [25, 26]. To date, most of the materials evaluated against readability are written at higher than the recommended reading grade level of 5th grade or lower. This is particularly important because given the reliance on the internet as a source of information among people with low health literacy level. Patients seeking information from the internet may find the information overwhelming and its usability low [29]. Furthermore, unlike people with a high health literacy level, people with low levels are unlikely to seek information from elsewhere [28]. Considering patients limited and inaccurate knowledge about HCAIs prevention and control measures and their limited health literacy [7, 11, 21, 30], patients are less likely to play an active role in HCAIs prevention and control strategies if the information is pitched beyond their reading level.

With regards to the video posted, the general approach was to embed information about infection preventing into a video covering a range of topics about hospital admission. In these cases, viewers were required to watch at least 8 min of the video before receiving any messages about infection control. Considering a recommended run time for those with low health literacy skills is less than 8 min [27], it would be worth evaluating how many people have watched the whole video clip and how much they can recall from it. Despite the availability of technology to support people with disabilities, reading difficulties or whose first language was not English with navigation, reading or translation of the materials, none of webpages offered or mentioned these resources.

Almost all the resources reviewed scored low in terms of cultural appropriateness. Most sites use generic ‘stock’ photos showing Caucasian doctors, nurses and patients, which does not represent the Australia healthcare workforce and does not reflect the staff members that they are going to interact with during their hospital stay. Seeing images dominated by Caucasian people and no mention of different cultural health beliefs or practices could make people from culturally and linguistically diverse backgrounds feel alienated and less receptive to the information being presented. Culturally appropriate and sensitive information can shape people’s health care experience in a positive way [31], increasing patient engagement in managing their care, as well as reducing healthcare disparities among different ethnic groups [32, 33]. Hospitals must consider the option of having resources available in the languages that represent the patients group frequenting the healthcare setting.

This study had several limitations. We only evaluated information from 19 of the public hospitals in Sydney (> 200 beds) out of a total of 121 (excluding dentistry, mental health and justice health). The findings from our study may not be generally applicable. Further studies are required to explore the delivery of online information in small/medium sized hospitals and in the private setting. Secondly, we only focused on the information available online and excluded brochures, posters, physical encounters with HCWs, etc. Therefore, determining the suitability of overall patient information available may be limited. One of main strengths of this study is that, to the author’s knowledge, this study is the first study assessing the suitability, readability and accessibility of online patient information on HCAIs available in Sydney. Secondly, despite the limitations, this study has identified some important issues regarding patient information relating to HCAIs.

## Conclusion

In conclusion, of the many factors influencing patient involvement and empowerment, the provision of information plays a critical role in reducing the burden of HCAIs. Current online patient information for HCAIs is too complex for patients to understand and act upon. Also, its accessibility and pitch currently limits the usability of the information. Further work is needed to address the appropriateness of hospital websites as platforms for promotion of infection prevention strategies and the receptiveness of patients towards receiving information in this format.
